# Multi-part quality evaluation of a customized mobile application for monitoring elderly patients with functional loss and helping caregivers

**DOI:** 10.1186/s12911-019-0839-3

**Published:** 2019-07-22

**Authors:** Matheus Costa Stutzel, Michel Pedro Filippo, Alexandre Sztajnberg, Rosa Maria E.M. da Costa, André da Silva Brites, Luciana Branco da Motta, Célia Pereira Caldas

**Affiliations:** 1 Universidade do Estado do Rio de Janeiro (UERJ), Rua São Francisco Xavier, 524 - Maracanã, Rio de Janeiro, 20550-900 Brazil; 2 Bacharelado em Ciência da Computação (BCC/IME) and Laboratório de Ciência da Computação (LCC), Rio de Janeiro, Brazil; 3 Programa de Pós-Graduação em Ciências Computacionais (CComp/IME), Rio de Janeiro, Brazil; 4 Programa de Pós-Graduação em Engenharia Eletrônica (PEL/FEN), Rio de Janeiro, Brazil; 5 Programa de Pós-Graduação em Ciências Médicas (PGCM/FCM), Rio de Janeiro, Brazil; 6 Núcleo de Atenção ao Idoso (NAI), Universidade Aberta da Terceira Idade (UnATI), Rio de Janeiro, Brazil

**Keywords:** Elderly monitoring, Caregiver support, Mobile application, Quality evaluation, Usability evaluation

## Abstract

**Background:**

The challenges faced by caregivers of the elderly with chronic diseases are always complex. In this context, mobile technologies have been used with promising results, but often have restricted functionality, or are either difficult to use or do not provide the necessary support to the caregiver - which leads to declining usage over time. Therefore, we developed the Mobile System for Elderly Monitoring, SMAI. The purpose of SMAI is to monitor patients with functional loss and to improve the support to caregivers’ communication with the health team professionals, informing them the data related to the patients’ daily lives, while providing the health team better tools.

**Method:**

SMAI is composed of mobile applications developed for the caregivers and health team, and a web portal that supports management activities. Caregivers use an Android application to send information and receive care advice and feedback from the health team. The system was constructed using a refinement stage approach. Each stage involved caregivers and the health team in prototype release-test-assessment-refinement cycles. SMAI was evaluated during 18 months. We studied which features were being used the most, and their use pattern throughout the week. We also studied the users’ qualitative perceptions. Finally, the caregiver application was also evaluated for usability.

**Results:**

SMAI functionalities showed to be *very useful* or *useful* to caregivers and health professionals. The Focus Group interviews reveled that among caregivers the use of the application gave them the sensation of being connected to the health team. The usability evaluation identified that the interface design and associated tasks were easy to use and the System Usability Scale, SUS, presented very good results.

**Conclusions:**

In general, the use of SMAI represented a positive change for the family caregivers and for the NAI health team. The overall qualitative results indicate that the approach used to construct the system was appropriate to achieve the objectives.

## Background

Elderly people with chronic diseases and functional dependence customarily demand long term and complex care usually provided by a caregiver. This is discussed, for instance, in the World Alzheimer Report 2016 published by Alzheimer’s Disease International, which points out the need for specific care pathways. These pathways include “training and support of primary care professionals by specialists and continuous care based on patients needs that change over time” [1].

As mentioned in the 2013 report by Alzheimer’s Disease International regarding the Americas [2], “the number of people with dementia in the Americas will nearly double every 20 years, increasing to 14.8 million in 2030, and 27.1 million by 2050. However, rates of increase through to 2050 will be much more rapid for Latin America and the Caribbean, than for North America. The increase in the number of people living with dementia will be most stark in low and middle income countries which will account for more than two thirds of cases by 2050.”

In the coming decades, the *aging index* is projected to substantially increase in Brazil: close to 20 by 2020, 28.5 from then to 2030, some 40 more between 2030 and 2040, eventually reaching 172.7 by 2050 [3]. This increasing aging index trend and the prevalence of chronic diseases among the elderly will most probably imply shifting patterns of morbidity and mortality.

In some developing countries, as in Brazil, it is not always possible to rely on professional care-giving: care is mostly provided by family members [2, 4, 5]. The family-relative caregiver is fundamental in organizing the caring routine for the elderly, which needs constant professional guidance and support, given the complexity and responsibility of this task [6].

Given this scenario, the Brazilian health care system has to be restructured in order to handle larger scale long-term chronic diseases, many of which are degenerative, possibly causing functional loss [5]. Thus, it is urgent to focus on developing solutions, within a feasible budget, which can enable health services to support family and caregivers in improved ways, helping care management.

Some recent studies have reviewed the outcomes of mobile technologies to help health professionals supporting and monitoring the elderly with chronic diseases and functional loss [7–10]. These studies also reveal that most applications and interventions do not have the caregiver as the main focus, which leads to declining usage over time, despite the verified potential benefits. Different from these works, our proposal applies mobile application technology focusing on the interaction between caregiver and health professional.

This paper presents the development and refinement stages, and an evaluation study of the Mobile System for Elderly Monitoring (*Sistema Móvel de Assistência ao Idoso*, SMAI). The general objective of this system is to improve daily care of elderly patients with dementia by improving caregiver-health professional communication. On the one hand, SMAI helps and supports caregivers managing care activities by making this interaction with the health team more fluid using a mobile application to send information to, and receive feedback from the health team. On the other hand, SMAI aims to empower the health team with timely and frequent information about the patients as a group, or individually, in a structured manner with a mobile application, helping the professional to work more closely with the caregivers.

One tested hypothesis is that the SMAI contributes to reducing the stress of caregivers, which expectedly results in better care administration and improvement to the elderly patient’s quality of life.

### Related work

There is a significant amount of interest in using telemedicine for controlling chronic diseases and for supporting health care systems [11–14]. In some of these studies the main point refers to the notifications and reminders to patients, in order to improve treatment adherence [13, 15].

Arif et al. [16] stress the importance of using specific technologies for telemonitoring and medical strategies to support the elderly living with chronic diseases. However, they emphasize that solutions must address the specific needs of this population in order to have a significant impact on improving their quality of life.

There are currently different health applications being used to monitor glycemia, blood pressure, daily exercise and nutritional support. But, after installing the software, many of these applications end up being discarded by the users due to usability problems or related to system instability [17]. According to Jin and Kim [18], most of these applications are developed with no previous requirements elicitation and with no clinical effectiveness assessed afterwards. In this context, Cook, Ellis and Hildebrand [19] emphasize that most of the mobile health applications are created without medical expert involvement and inaccurate content, resulting in risk of harm to the patient.

Systematic reviews regarding technologies to assist older people and the aging population have verified the factors that influence acceptance [20], highlighting the acceptance difference regarding pre- and post-implementation, and the barriers to its adoption [21, 22]. The analysis of systems focusing on chronic diseases selected from the systematic review developed by Khosravi et al. [23] has results that range from “no effect”, in one of the systems, to “increased quality of life” and “social functioning” or “decrease in the number of hospital readmissions” in most of the systems.

In that regard, Nicholas et al. [24] evaluated different e-Health technologies. Several reasons have been identified as recurring problems related to the discontinuation in using the e-Health system: users do not receive sufficient incentives, loss of interest and weak perception of real benefits, and other reactions. More recently Lee et al. [25] reviewed behavioral intervention strategies using mobile applications for chronic disease management. Their work found that features such as text reminders and improved communication between patients and healthcare providers result in “enhanced self-management in patients with chronic conditions”. However the relation engagement of users and outcome improvements were not conclusive.

## Methods

### Setting

SMAI was co-designed by researchers from the LCC - Computer Science Laboratory (*Laboratório de Ciência da Computação*) and health professionals from the NAI - Care Center for the Elderly (*Núcleo de Atenção ao Idoso*) at the Rio de Janeiro State University (UERJ). NAI maintains a multidisciplinary health service for elderly patients that present different levels of neurocognitive disorders (dementia). A team composed of geriatricians, physiotherapist, nurses, social service professionals, nutritionists and psychologists assists over 250 patients and their caregivers. The team is prepared to maintain the service, also taking into account commonly occurring adverse characteristics: (i) the caregiver is a relative of the patient, often under stressful situations due to his/her caring activities, (ii) on average, these are low-income families, mostly residing in peripheral neighborhoods, and (iii) most of them use public transportation to get to NAI. On-site consultations result in the caregiver and the elderly patient having to make sacrifices.

The study comprising SMAI assessments and a quality evaluation was conducted by ten professionals from the NAI health team. The target of the system was a group of patients in different stages of dementia, with functional loss and limited autonomy, which are supported by caregivers. The caregivers engaged in the project in the early stages as alpha testers, with informal feedback, and participating in the formal assessments focus groups, retraining meetings and usability evaluation. Thus, the design of SMAI considers two main actors: the caregiver and the health professional. The study and the consent to collect and use data information from participants was reviewed and approved by the Research Ethics Committee of Pedro Ernesto University Hospital (UERJ Hospital’s Institutional Review Board), CAAE number: 32654014.9.0000.5259.

### Project stages

**Fig. 1:**
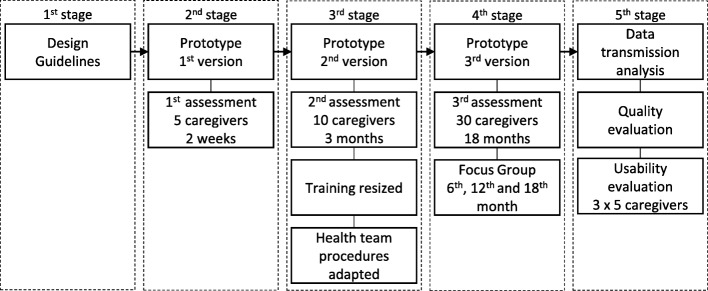
Project stages

Figure 1 presents the flow of activities of the project, while contrasting concerns and impressions of the group of participating caregivers and the health professional team.

The initial stage of the project started in early 2014, defining the design guidelines, which should be driven by the expertise and clinical practices of the NAI health team, and the mobile application requirements applied for health support. Subsequently, a first prototype was built.

Two initial short assessments were performed. The first, carried out with five caregivers, verified how the first prototype would behave. These five caregivers would be alpha testers, using the prototype under real conditions. There were many variables to consider: from the correct execution of the application, to access of the remote database. In addition, a brand new smartphone was being handed to the participant caregivers, with a recently registered prepaid data SIM card with limited credit. After two weeks the caregivers returned with comments and operational issues, which led to improvements in the prototype.

The second assessment was carried out with a group of 10 caregivers, who received 40 min of individualized training and then used SMAI for three months. The feedback from the caregivers was very insightful and allowed the computing team to make many other improvements in the applications. It also allowed the health team to revise their procedures.

The refined system allowed performing a broader assessment. This time, a qualitative-quantitative mixed clinical evaluation with a group of about 30 caregivers using SMAI for 18 months was carried out by the health team. The objective was evaluating the caregiver’s perception regarding the use of the system as an auxiliary tool, and the experience of the health team monitoring the group with the system. Focus Group interviews were scheduled every 6 months and a final questionnaire was responded by both the caregivers and the health team.

Also, during the clinical evaluation, a “users’ impression” evaluation and a formal usability evaluation were also carried out.

### Design guidelines

In October 2013, before other project activities, a survey questionnaire was responded by 47 volunteers. Of the total of respondents: 94% (44 users) used mobile phones and 77% had computers at home; 72% were used to sending short messages (SMS); 80%, or someone close, used e-mail and/or had social network accounts; 72% had Internet access at home. Access to the Internet was already part of their daily life: 86% used the Internet several times a week, and 40% every day.

The Android system was chosen as the base for the mobile applications given that Android-devices are often inexpensive and yet sufficiently powerful. Furthermore, Android-devices were prevalent among the surveyed volunteers, although many of them had devices running vendor-specific proprietary systems or that were not considered “smart”.

Joint meetings involving the technical and health teams were scheduled to sort out the guidelines for the software system. As a general strategy, it should be customized considering the profile of the caregivers eligible to use the system, limitations of this group, and the current clinical and care practices, as well as the experience of the team monitoring this group.

#### General requirements

One general requirement was to have a design solution that should be simple to use without requiring special skills, long training or complex deployment. Using ambient sensors or wearable devices to capture physiological measurements in a smart setting was discarded. In addition to having pros-and-cons [26], simplicity and low cost was a prerequisite. It would not be affordable to deploy a complex system, with sensors and processing nodes, in a reasonable number of homes of the group of patients.

The system was structured with mobile applications for smartphones (caregivers) and tablet devices (NAI team), and a Web portal supporting management activities. All data is sent to and received from a cloud application server.

As basic requirements, the caregiver’s application would be used to send information and patient reports to the health team. It should also be prepared to receive data from the health team and display it as notifications, messages or scheduled appointments. These basic requirements would allow the caregivers to maintain their habitual care routine while being better connected to the health team with an absorbable overhead.

The health team application should allow each professional to easily visualize the group of patients and to access individual data. Data received from all caregivers should be shared among all health professionals. It should also support configuring individual notifications and appointments for a patient, as well as sending and receiving individual or group text messages to the caregivers. A response, message or notification configured by a health professional should be registered and traceable.

#### Specific requirements

The health team involved in this phase of the project also had further concerns regarding non-functional aspects, recurrently required for distributed eHealth applications: privacy, security and fault-tolerance to network unavailability.

Each user should have a unique identification in the system and should be authenticated before sending and transmitting data. All communication and data persistence should be encrypted to guarantee authenticity and confidentiality. Access control mechanisms should mediate the access of the health professional to guarantee data sharing authorization within the application server. All communication sent by the health team should be labeled with the ID of the respective professional.

The applications should be tolerant to network failure and Internet unavailability. From the user’s point of view, the system should behave as always connected. The mechanisms to mitigate these problems should maintain robustness and reliability, timestamping and labeling every message, and using retransmission mechanisms. These items were considered at design time.

As an additional point, based on the adopted practice of the service offered by NAI, and supported in works such as [27] the application should also be playful, with gamification aspects. This would contribute to increasing the caregiver’s interest to continuously use the application.

The system was designed and implemented considering all elicited requirements. Stutzel et al. present a broad discussion on SMAI architecture, non-functional requirements and on gamification aspects introduced in SMAI [28].

## SMAI overview

**Fig. 2:**
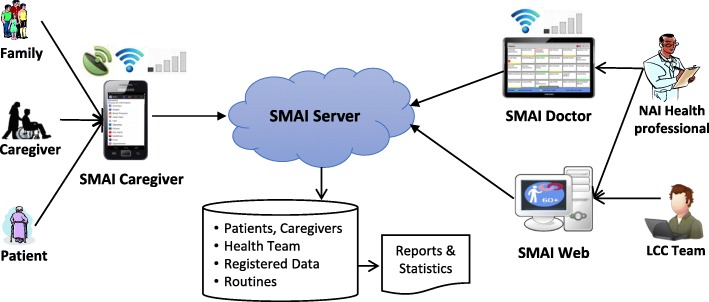
SMAI overall structure. NAI: Care Center for the Elderly (*Núcleo de Atenção ao Idoso*); LCC: Computer Science Laboratory (*Laboratório de Ciência da Computação*); SMAI Caregiver, SMAI Doctor and SMAI Web: applications that compose SMAI

The Mobile System for Elderly Monitoring, (*Sistema Móvel de Assistência ao Idoso*, SMAI), is composed of two Android mobile applications (*SMAI Caregiver* and *SMAI Doctor*), a Web application (*SMAI Web*) and an application server (*SMAI Server*) as a front-end to a database (Fig. 2).

### SMAI caregiver interface

**Fig. 3:**
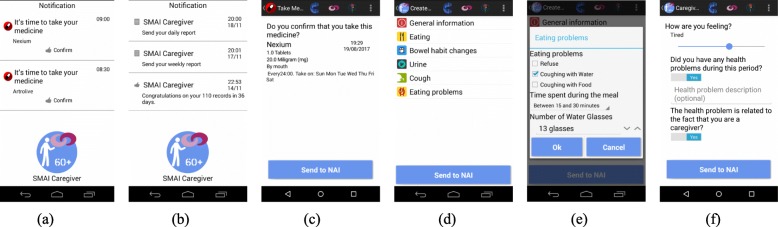
SMAI Caregiver interface. **a**, **b** and **c** Panel of Notifications and Reminders. **d**, **e** and **f** Patient and Caregiver Reports. Patient Reports are sent every day, while Caregiver Reports are sent once a week

Caregivers carry an Android smartphone running SMAI Caregiver. The application allows the caregiver, on behalf of the patient, to receive notifications and reminders anytime from NAI health team about time to take medications (Fig. 3a), appointments or to send a report (Fig. 3b). Medication reminders have to be confirmed (Fig. 3c). The caregiver is notified to fill the Patient Report every day at 8:00 PM (Fig. 3d) with specific information (for example, Fig. 3e). A short Caregiver Report also has to be responded once a week (Fig. 3f) allowing the NAI team to also monitor the caregiver’s stress level.

**Fig. 4:**
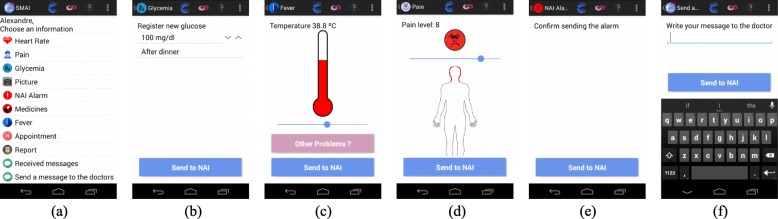
SMAI Caregiver interface. **a** Main Options and **b** to **f** Input Panels

The application also allows sending physiological information, photos and text messages at the caregiver’s discretion. Beginning at the main options menu (Fig. 4a) the caregiver can easily send information about Blood Pressure, Glycemia (Fig. 4b), Temperature (Fig. 4c) or Pain (Fig. 4d), for example. In addition, the NAI Alarm *button* can be used to report an emergency situation to the NAI team (Fig. 4e), accompanied by a text message (Fig. 4f).

### SMAI Doctor interface

**Fig. 5:**
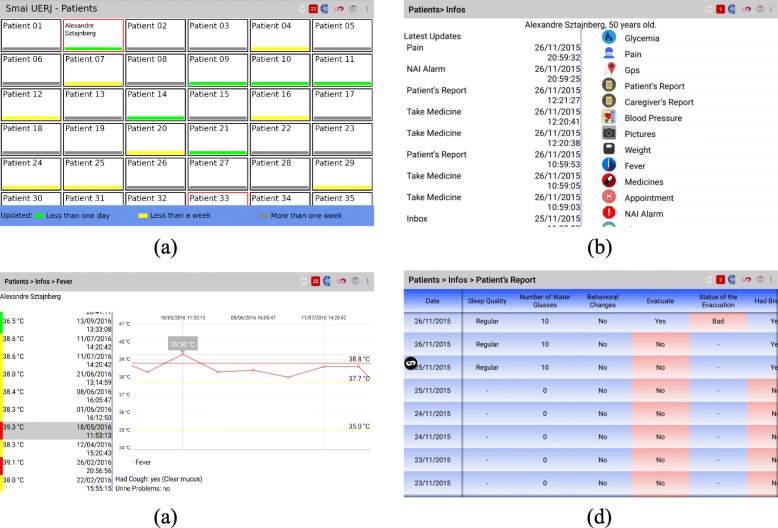
SMAI Doctor interface. **a** Dash Board. **b** Patient details. **c** Temperature History and Details **d** Patient Daily Report

The NAI health professional involved in the project has an Android tablet running the SMAI Doctor application. A Dash Board displays all caregivers interaction status (green indicates that some information was sent over the last two days, while gray indicates that no information was sent in the last week), and highlights, with a red margin, that the patient has sent a NAI Alarm requiring attention (Fig. 5a).

Previously sent information can be browsed by clicking a patient’s button (Fig. 5b), including history data and reports. For example, Fig. 5c shows Temperature history details, while Fig. 5d shows the interface for the Patient Report.

For each patient the professional can configure notifications and reminders and also send text messages.

## Evaluation methods

Challenges in evaluating digital interventions, such as SMAI, range from the patients’ variable *engagement* to the difficulty of measuring *effectiveness* [29]. One of the difficulties pointed out is how to specify comparison interventions or control conditions due to the lack of comparable parameters. Therefore, we combined some objective and subjective multi-part assessments to evaluate the system.

As described in Fig. 1, the first working prototype of the SMAI system was submitted to two short preliminary assessments (Stage 2 and Stage 3). After the prototype refinements, a long-term clinical evaluation was carried out (Stage 4), along with a general user’s impression survey and usability evaluation (Stage 5).

### Preliminary assessments

For the *first assessment*, a small group of 5 caregivers assisted by the NAI team received a Motorola Moto G smartphone pre-loaded with the first version of the SMAI Caregiver prototype and with Internet connection. The group was briefed on how to use the application.

At this stage (Stage 2), the objective was to have the caregiver’s overall perception about the system, if the navigation was easy to use and about the caregiver’s overall willingness and perseverance concerning the system. Each caregiver should freely explore the new tool for two weeks.

The two project teams monitored the caregivers. The health team used SMAI Doctor to keep track of the sent information and location data, which the SMAI Caregiver also transmits. The LCC computing team monitored network and data traffic counters using the SMAI Web application.

A *second assessment* was then carried out with a group of 10 caregivers, randomly selected, who received training and then used SMAI for three months (Stage 3). The hands-on, 40 min training was individualized. Each of the application’s functions was explained and demonstrated. Then, the caregiver was asked to test the system herself, and perform some simple tasks suggested by the health professional accompanying the execution. The remaining doubts were resolved at this time, and if new doubts arose during the daily use of the system, a new training was done at the next patient visit to the NAI service.

### Clinical qualitative evaluation

A broader long-term assessment was carried out (Stage 4) with a larger group of caregivers considering a qualitative quantitative mixed clinical intervention. This step also involved the health team. The aim was to evaluate how SMAI was perceived by the system users in their routines, considering the group of caregivers:

- how the group used the mobile application; if it was useful; and how it affected patients’ and caregivers’ daily activities;
- if the use of the system brought improvement to care activities; if it was easier to deal with new caring situations;

and regarding the health team:

- how the health professionals perceived the new application regarding their clinical practices.
- if the tool was useful; if it helped monitoring a group of caregivers.

The smartphones with pre-loaded SMAI application were assigned to caregivers using convenience sampling. As inclusion criteria the caregiver should be assisted by NAI, in this specific clinic, as well as having some skills using a smartphone. Qualitative data would be collected through Focus Group interviews.

#### Focus group with caregivers

The Focus Group interviews allowed capturing the caregivers’ perception regarding the use of the SMAI Caregiver application. Three meetings were held with the caregivers at 6, 12 and 18 months of use. All interviews were audio-recorded and the content was analyzed and categorized. Due to the already discussed difficulties related to mobility and transportation (quality of public transport and distance from home to NAI) and the ever-complicated problem of leaving the patient with another caregiver or relative, participation in the group sessions was not homogeneous.

#### Focus group with health professionals

The health professionals’ perception on the use of the SMAI Doctor application was evaluated in a single meeting, 18 months after the beginning of the intervention. Positive and negative aspects of the system were raised, as well as modification suggestions, including solutions to the identified negative points. Based on the content analysis, the following thematic categories were highlighted:

- “the SMAI Medical application and my professional practice”;
- “perceptions about the SMAI Medical application” and;
- “solutions and ideas for the caregivers application”.

### Quantitative observations

To complement the qualitative results verified, in relation to the caregivers, some quantitative information was assembled by mining the collected database. The database represents 96 weeks (22 months), regarding Stages 2 to 5. An expected outcome of the project was to understand the caregivers’ involvement, whether she/he was a very participative user or not, if specific use patterns could be identified and how the use pattern of the system would change in a long-term run.

### User quality/satisfaction evaluation

During the last stage of the project (Stage 5), an evaluation was also carried out with the participants of the two groups of users through a qualitative/satisfaction survey with Marked Semantic Differential Scale [30] responses. The objective was to verify, in addition to the data retrieved from the database, the impressions about the application and which functionalities were most important for each group: caregivers and health professionals.

The survey was divided in two parts. The first, with statements regarding how the participant perceived the SMAI features, with pre-defined options spanning form “very useful” to “not useful” or “didn’t understand”. The results are discussed with the help of plots with the percentage distribution among the options (rounded to the nearest whole number).

The second part included statements on how the participant perceived SMAI in her/his daily activities and the general sense of satisfaction, which should be ranked from “strongly disagree” and “disagree”, with a “neutral” rank, to “agree” and “strongly agree”. The five adjacent response options were assigned a 5-point ordinal scale. For each statement the result is presented with the response frequency, along with the weighted mean, standard deviation, and the upper and lower bounds for a 95% confidence interval considering a Student’s t-distribution.

The designed statements were related to positive aspects of the intervention, with the exception for “stress”. As discussed in the Introduction section, one of the objectives of our research was to assess whether the proposed system would introduce or increase the level of stress of caregivers and health team professionals. The stress-related statement followed the same style as the other assertions, in a direct way. Thus, unlike the other responses, it was desired, in this case, that the responses should be “disagree”-bound, indicating that the system did not introduce stress.

### Usability evaluation

In general, usability is evaluated by observing how the users interact with the software, considering *task completeness*, *completion time*, and *learnability* metrics [31]. The International Organization for Standardization, (ISO 9241—11, 1998, p.2) [32] defines usability as “the extent to which a product can be used by specified users to achieve specified goals with effectiveness, efficiency and satisfaction in a specified context of use”.

Our approach was to evaluate usability in two steps: the first one closely followed the evaluation model proposed by Jakob Nielsen [33], in which users are observed, to verify the ease of use, while executing a list of activities; the second one applied the System Usability Scale, SUS [34], to have a single score to classify user satisfaction with SMAI.

The number of participants was determined based on studies, which state that the best results are obtained with five users of the same profile, justifying that after the observation of the fifth user the collected behaviors are similar [35]. Fifteen caregivers were selected, 11 female and 4 male caregivers, with the daily use of smartphones as the inclusion criterion. The 15 participants were distributed in three groups of five participants:

- *Current SMAI user*: have been using the application for some time;
- *New user, with training*: had no experience with the application, and received rapid training on the application’s functionalities;
- *New user, no training*: had no experience with the application, and did not go through the training stage.

The strategy of using 3 groups of users, with 5 participants each, allowed to broaden the evaluation of the system in three aspects:

- Memorizing the execution of tasks for the user who had already used the system, to verify ease of use.
- Easy task execution for the user who had not used the system and who would receive a brief explanation about the operation of SMAI, to verify if the system is easy to learn.
- Facility in the execution of tasks for the user who had not used the system and who would not receive explanation about the operation of SMAI, to verify if the system navigation is intuitive.

**User Observation.** After a brief presentation of the assessment routine, and after signing the consent form, eight tasks, in ascending order of difficulty (classified by the expected number of steps to be performed) were proposed to each participant:

1. Confirm that the medication was given to the patient;
2. Tell the doctor about the patient’s conditions using the text message;
3. Send activity information;
4. Send blood glucose measurement;
5. Send the requested information;
6. Send an alarm;
7. Respond the notification to send weekly caregiver report;
8. Respond the notification to send the patient’s daily report.

All steps were recorded on the smartphone using the free version of the AZ Screen Recorder software, available at Google Play [36]. The recorded data generated approximately 5 h of videos. They were analyzed to code the observed events: completion of tasks, number of steps used and execution time of each task. Additional information on each participant, including oral communication, was also noted.

**SUS survey.** For the second usability evaluation step, we studied several usability evaluation models that consider different usability issues [37–39]. Based on the characteristics of the participant caregivers (age and years of education, Table 1), the low technology literacy observed during the clinical evaluation (Stage 4) and results presented in [40] and [41], we considered adopting a simple and accurate quantitative tool to rate the SMAI usability level. The System Usability Scale (SUS) developed by Brooke [34], can quickly and easily collect a user’s subjective rating of a product by providing a single score that classifies the quality level of this product. Venson et al. [42] stress that it is scientifically accurate and is not excessively long to the user. According to Bangor et al. [43] the SUS is an inexpensive and effective tool for assessing the usability of smartphone applications. SUS presents 10 statements related to a 5-level scale of agreement. It can reach a maximum score of 100 points, where higher scores indicate better usability.

**Table 1:** Demographic characteristics of the participant caregivers

Characteristic	Caregiver (*N*=38)
*Age* 61 years (avg)	SD = 10.75
*Gender*	
Female	32(84%)
Male	6(16%)
*Status*	
Married	20(53%)
Widowed	3(8%)
Divorced	5(13%)
Single	10(26%)
*Education*	
≤ 12 years	21(65%)
> 12 years	17(45%)
*Relationship* (with the patient)	
Children	29(76%)
Partner	7(18%)
Other	2(6%)
*Occupation*	
Employed	7(19%)
Unemployed	4(10%)
Retired	27(71%)
*Family income* (minimum wages)	
≤ 2 MW	10(26%)
2–4 MW	23(61%)
4–6 MW	5(13%)
> 10 MW	0(0%)

Thus, after completing the previous sequence tasks, all 15 participants involved in the usability evaluation process also answered a survey consisting of 10 statements about the use of SMAI, allowing to compare the previous observations with the participant’s impressions. The collected data was organized and evaluated.

### Characteristics and clinical staging

Tables 1 and 2 summarize a profile survey of the demographic and specific characteristics of the caregivers. It was observed that the participants themselves can be considered elderly, on average 61 years of age, represent the female gender (84%), married (53%), have over 12 years of education (45%) and moderate (37%) or moderate to severe (37%) level of stress and overload. They are children of the patient (76%), retired (71%), have a family income of 2 to 4 minimum wages (61%) — low purchasing power, they have cared for the elderly for up to 5 years (53%), they share the care responsibility with another person (55%), they live with the elderly (82%), they have 1 to 2 diseases (58%) and they are not under psychological support or monitoring (79%).

**Table 2:** Specific characteristics of the participant caregivers

Characteristic	Caregiver (*N*=38)
*Time as caregiver*	
≤ 5 years	20(53%)
6–7 years	7(18%)
8–10 years	7(18%)
> 10 years	4(11%)
*Sharing care (with other person)*	
Sharing	21(55%)
Not sharing	17(45%)
*Residence with the elder*	
In the same house	31(82%)
Other house	7(18%)
*Formal caregiver*	
Yes	10(26%)
No	28(74%)
*Stress/overload* (Zarit scale)	
Moderate	14(37%)
Moderate/Severe	14(37%)
Severe	3(8%)
Low/No	7(18%)
*Reported health problems*	
No problems reported	3(8%)
1–2	22(58%)
> 2	13(34%)
*Psychological support*	
Yes	8(21%)
No	30(79%)

It is important to add that 82% of the family caregivers exhibit some level of overload and only 26% of the elderly had a formal or paid caregiver.

The elderly patients eligible to participate in the study were also surveyed (general profile in Table 3 and clinical staging in Table 4). They are 84 years old on average (SD =7.14), female (79%), widowed (71%), have 8 years of basic education (27%), have severe disability (82%), impaired mobility (42%) and Alzheimer’s disease as the main diagnosis (71%). Concerning the stage of dementia, 53% of the patients have moderately severe dementia, followed by severe dementia (24%).

**Table 3:** Demographic characteristics of the elderly patients eligible to participate

Characteristic	Patient (*N*=38)
*Age* 84 years (avg)	SD = 7.14
*Gender*	
Female	30(79%)
Male	8(21%)
*Status*	
Married	10(26%)
Widowed	27(71%)
Single	1(3%)
*Basic education*	
Illiterate	8(21%)
≤ 8 years	27(71%)
9–11 years	3(8%)

**Table 4:** Clinical staging of the elderly patients eligible to participate

Characteristic	Patient (*N*=38)
*Functional disability*	
Moderate	7(18%)
Severe	31(82%)
*Mobility*	
Impaired	16(42%)
Not impaired	22(58%)
*Main diagnosis*	
Alzheimer	27(71%)
Other dementia	11(29%)
*Dementia stage (FAST scale)*	
Mild	4(10%)
Moderate	5(13%)
Moderately severe	20(53%)
Severe	9(24%)

The initial number of participants was 38 caregivers. However, 10 participants were excluded during the study: five due to dropout, one because the elderly participant was institutionalized, one due to theft of the smartphone and three due to the death of the patient. Twenty eight participants remained in the investigation.

## Results

### Preliminary assessments

In the first assessment, after fifteen days of use, the system registers indicated that the group of five caregivers had used the system every day, sending data at a variable pace. On average, 700 Kb was transmitted every day per caregiver.

Also, the group was informally interviewed. The caregivers pointed out positive and negative aspects — reporting that the system was usable and they would use it in the long-term. But, there were some problems understanding certain features, such as the *Physical Activity*, and the notification-menu flow. With this feedback, some interface panels were improved in a new prototype. The observations also signaled the project team that a more complete training was necessary.

Regarding the second assessment, after the first month using SMAI, a collective retraining with the ten caregivers was scheduled; and after the third month a *Focus Group* interview [44] concluded the assessment.

The feedback from the caregivers allowed the computing team to make many improvements in the applications. The interface was reorganized to guide some interactions, two report items were introduced in the caregiver’s report (for instance, asking if the caregiver herself had any health problems that could be related to caregiving activities) and some changes were also implemented in the notification engine. The version of the system resulting from this second round of refinements is the one presented in this paper.

In addition to the improvements made on the application, this second assessment also allowed the health team to revise their procedures. A result worth mentioning was the proposal of new operational protocols given the different possible interactions introduced with the use of the system. One of the issues raised was the activation of the NAI Alarm (see Fig. 4e). The caregiver had to be informed that the NAI Alarm is not a call to an emergency service, and that a NAI Alarm should be accompanied by an explanation (Fig. 4f). It should be used as a mechanism to notify the NAI team regarding an important event that has occurred with the patient, but the caregiver should not wait for a response and should take the patient to an emergency service as soon as possible.

Another point discussed in this context was how the NAI team should handle the alarms and message notifications, how timely and in what manner they should be responded. Some of the ideal solutions raised were organizing health team shifts, or deploying a 24/7 monitoring service, which could initiate a first level intervention. However, given that NAI offers ambulatory services, making it impossible to have emergency service and night shifts, the feasible trade off was to have NAI health professionals voluntarily take turns at night and on weekends to monitor alarm notifications, acknowledge the alarm with NAI Doctor (making the team aware) and reply to the caregiver as soon as possible.

### Clinical qualitative evaluation

#### Focus group with caregivers

From the content analysis of the Focus Group session at 6 months, themes related to *communication* and *medication management* emerged. The most commonly highlighted positive aspects were:

- the system helped the caregivers maintain connection with the NAI team, and
- the medication notification helped correct the medication administration.

The Focus Group at 12 months discussed themes related to:

- the patient’s management strategies,
- being a caregiver and a member of the family, and
- the caregiver’s illness.

This second session captured more personal and specific points affecting the caregiver, helping the mediator confirm some of the important aspects the project tries to assist.

After 18 months the mediator focused on evaluating the application using previously prepared questions. As a result of this final evaluation, the positive impact on daily care was highlighted, *reducing the need for locomotion to seek professional guidance*, which increased the caregiver’s confidence.

#### Focus group with health professionals

As an overall result of the Focus Group session, the health team found that the system improved the monitoring of patients and that it also facilitated interacting with the caregivers. Nevertheless, the most discussed aspects revolved around the effect the system had on the professional’s daily practice, also compared to the positive aspects in the monitoring of patients. This issue also appeared in the quality/satisfaction evaluation results. Among the improvement suggestions in this regard, there was a proposal to simplify the report module to avoid having to fill out a detailed report every day. The idea behind this is to minimize the caregiver’s workload.

### Quantitative observations

**Fig. 6:**
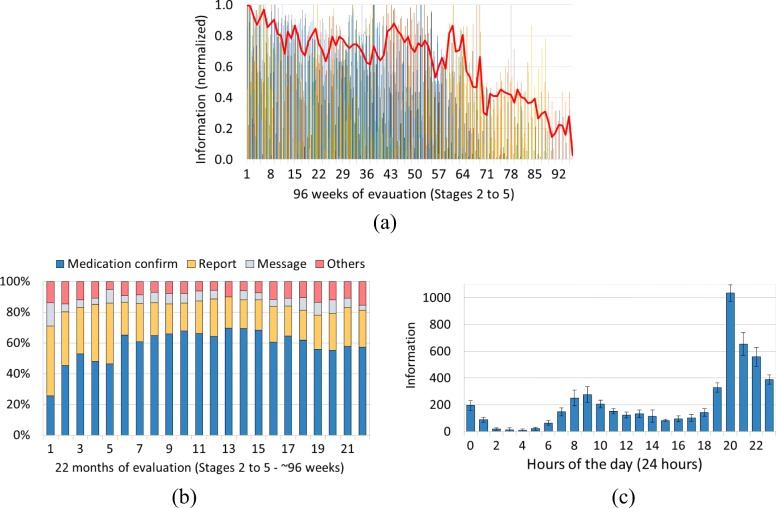
Quantitative data analysis graphs. **a** Amount of information sent (normalized). Individual (multi-color) and average (in red) normalized quantity of information sent by the caregivers during the 96 weeks SMAI was used. **b** Information sent by type. Frequency distribution of information type sent by the caregivers during the 22 months (96 weeks) SMAI was used. **c** Information sent by hour. Average amount of information sent by caregivers hourly every day (24 h)

Figure 6a shows the caregiver data transmission profile from the first day of use until leaving the project. To provide more information, the amount of data sent by each user is also presented, along with the averages. The first week of use for each caregiver is plotted at point 1 of the x-axis (first week) regardless of when their participation date actually began. The amount of information sent was normalized: a value of 1 means the maximum amount of information that the caregiver sent at any week during her/his participation, regardless of the type of information sent. It can be observed that on average the caregivers sent larger amounts of data at the beginning of their participation, stabilizing between weeks 11 and 41, with this pattern then decreasing over time. On the other hand, it was also seen that several caregivers have maintained an above average reporting pattern.

An average of 416 pieces of information was sent per week throughout the project. Here, one piece of information considered is the data transmitted from any SMAI Caregiver input module or report panel. Whether sending the patient’s temperature, a report or an image, which have different sizes, the action accounts for one piece of information. During the 96 weeks of operation, which comprised Stages 2 to 5, the system registered an average of 1644 pieces of information sent manually by each caregiver and 6958 pieces of location information sent automatically. A total of 72,375 pieces of information was sent directly by the group of caregivers and a total of 334,014 pieces of positioning information was registered in the database.

We also consolidated the data regarding the features and times they most used the system. Figure 6b shows how the available features were used each month. The medication confirmation mechanism and the completion of the daily report were the most used, collectively representing an average of more than 60% of the information sent per month during the analyzed period. It can be observed that in the first months, when the health team was adapting the new routines, medication confirmation was less used. This feature needs a health professional to input the medication, the doses and times when it has to be administrated to the patient. In addition, we emphasize that from August to November 2016 (months 12 to 15) the sum of information sent regarding these two features reached 90% of the total recorded by the system. This shows that mechanisms that generate some kind of visual or audible notification induced the caregiver to interact with the medical staff through SMAI. Among the features without an associated notification mechanism, sending text messages was the most used.

The time span the system was most used throughout the day, regardless of the day of the week, are 8:00–9:30AM and 8:00–9:00PM, correlated, once again, with the time when most medication notifications were configured by the health team and with the notification to fill the daily report, respectively (Fig. 6c). There was also some intense use in the time range between 9:00–11:00PM when patients are usually asleep and caregivers were able to use the system to send the report and other supplementary information.

### User quality/satisfaction evaluation

**Fig. 7:**
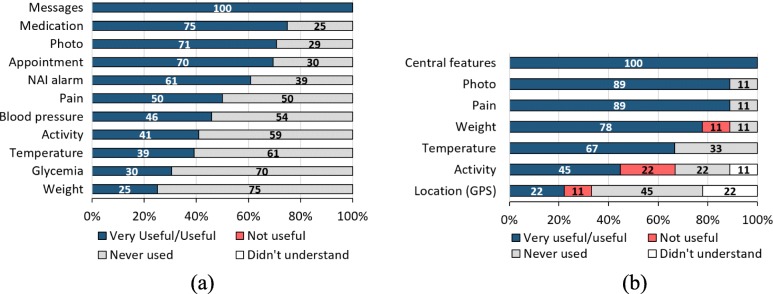
Survey on how **a** caregivers and **b** health team perceived the features most used. Bars represent the frequency distribution of pre-defined responses (% rounded to the nearest whole number)

**Caregiver Group:** (24 respondents from 28 participants, *N*=24). Among the points raised, caregivers were asked which features were the most useful in addition to the daily report. Messages to the health team were considered as very useful or useful by 100% of the caregivers (Fig. 7a). We observed in the database that quite frequently the interaction of the caregiver with the NAI team by means of exchanging text messages was very reassuring. Notification and confirmation of medication was also considered useful (75%) and, at a similar level, sending *Pain* information, patient pictures (*Photo*) and appointment scheduling (*Appointment*) was considered as useful or very useful by more than 50% of caregivers. Also, the NAI alarm can be highlighted as very useful, given that, like the features mentioned above, it reached equally positive values, approximately 61% of approval. The other available features were found to be useful to a lesser extent. *Glycemia* and *Weight*, for example, were not used by 70% and 75% of caregivers, respectively.

The evaluation of the caregiver group, containing qualitative questions with Likert [45] scale responses, was very positive (Table 5). The overall application satisfaction related to using the system was high: 15(63%) caregivers were satisfied and 8(33%) were fully satisfied, with a mean score of 4.29. Most caregivers, who accounted for 84% of the total (20 out of 24), disagreed or strongly disagreed that using the system had increased their stress, with an expected low score mean (1.83). Some points were evidenced from this evaluation, some of which suggests that the use of the application helped positively in their patient care, with a 4.25 mean score, which had an approval of 19(79%) caregivers. SMAI improved the day-to-day of the caregivers by facilitating their activities, since information about prescribed medications, scheduled appointments and messages could be easily viewed.

**Table 5:** Survey on quality impressions of the system perceived by the caregivers

	

The application also helped to connect and simplify communication between health professionals and caregivers, giving the caregiver the sense she or he was always connected to the health care professional (mean score of 4.42). As a last point, as mentioned earlier, *gamification* aspects were introduced in the application. Based on a simple count of transmitted data the caregiver received a congratulatory message. This feature had a positive effect on using the system for a significant number of caregivers (14(58%) agree and 7(29%) strongly agree, with a mean score of 4.17).

**Health team:** (9 respondents from 10 participants, *N*=9). In general, the system’s functionalities received positive feedback from the health team (Fig. 7b). Among them, the central features, such as patient and caregiver *Reports* are highlighted, as well as message exchange, medication prescription, and sending Blood Pressure, Glycemia and NAI Alarm, all showing an acceptance of 100%.

The other functionalities were also ranked as useful or very useful, with most having an acceptance level of more than 60% of the health team.

Some features, however, were not deemed useful and need to be re-evaluated. *Activity* information, for example, was not rated as useful. In fact, it had already been found that the options for the caregiver to inform the patient’s activities were far from reality. For example, *running* or *swimming* were two of the options, which should be accompanied by time and distance traveled. There was the case of a caregiver who had the patience to introduce activities such as “dish-washing”, “walking” or “gardening”, and then selected these activities to be reported. The development team had also anticipated that the *Location* feature would have limited use. This requirement had been introduced by the NAI team in order to locate the patient, who could get lost in several situations or, as a second possibility, the patient’s mobility patterns could be assessed. However, it cannot be taken for granted that the device will always follow the patient’s localization. In many situations, the device was left in the residence while the patient was moving or the caregiver had taken the device without accompanying the patient.

In the quality evaluation (Table 6), mean scores above 3.78 for most statements, suggest that professionals agree that the system achieves the objectives of improving the quality of life of patients and caregivers. Driven by the notifications sent by SMAI, namely alarms, confirmations and reports sent out by caregivers, the staff managed to provide continued attention without the limitation of having to wait for the next appointment.

**Table 6:** Survey on quality impressions of the system perceived by the NAI health team

	

One result worth discussing in the quality evaluation, also mentioned in the clinical qualitative evaluation, is related to the last 2 questions regarding the Health Team (Table 6). Four health professionals (over 40%) found that using the system was stressful (3.22 mean score contrasting with an expected low score) and only 5(44%) (3.78 mean score) would use the system in their daily clinical practice. Still, they would recommend the adoption of SMAI (100%, with a mean score of 4.56). It is observed that during the Focus Group meeting with the professionals, it was unanimously mentioned that using the system brought more responsibility to the team, many of whom were resident physicians, which now shared the responsibility to “continuously” monitor the patients, evaluate the information sent and respond in some way, in addition to the traditional face-to-face appointment time. For that reason, the system was considered stressful by a significant percentage of professionals (44%), although they perceived its positive qualities. It was argued that the system should be operated by a supervising health team with a follow-up system that could filter the information that required the health team’s attention, and perform the first interactions with the patient.

Another point that should be considered, in this context, is that despite many of the specific requirements that were proposed by the health team, many of the core characteristics were proposed by the NAI team supervisors’ subgroup. Some requirements led to making immediate adjustments on the application as soon as the first version of the system was released, but many of them required, as mentioned earlier, making adjustments on the operational routine. Lastly, there was a certain turnover of the health team. The group of professionals that were engaged in the project at the beginning, when many of the requirements were designed, was not the same group as when the 18 month evaluation started. These points help understanding the stress increase.

### Usability evaluation

**User Observation.** The usability evaluation results helped us observe that even with no previous training, the *New user, no training* group was able to perform the tasks with a good rate of success. The *New user, with training* group, which received a short training prior to the evaluation, was the one that performed best, outperforming the *Current SMAI user* group, which may indicate the need for periodic retraining to refresh the use of some features and that the application has a simple interface, reinforcing the obtained above average result by the third group (*New user, no training*).

**SUS survey.** The average SUS score found and the standard deviation, SDV, are presented in Table 7 for individual groups and globally.

**Table 7:** Usability evaluation SUS score

Group	SUS score	SDV
*Current SMAI user*	86.25	10.09
*New user with training*	68.00	17.45
*New user, no training*	78.00	16.34
Global	77.42	9.14

The global SUS score found was 77.42, with a standard deviation of 9.14, which, according to the scale presented by Bangor [43], indicates that the application can be ranked in the range between “good” and “excellent” levels of usability.

The SUS survey results were also aligned with the performance of executing the tasks of the first usability step. The participants considered the application was easy to use and consistent. However, there were some improvement demands on the data input panels (which can be updated with the most recent Android standard interface). Also, the *New user, with training* group participants mentioned that the short training made the difference, while in general the third group participants, which had no training, mentioned that the application was easy to use but it would be easier if they had previous training. This is reflected in the standard deviation and suggests that the SMAI users could feel more secure using it after receiving training, which is already being done. In addition to training, the system could rely on a help module, using explanatory videos, to help users better understand how to use each of the functions of the system.

## Discussion

Contrary to what was observed in Jin and Kim [18] and Cook, Ellis and Hildebrand [19] studies about the lack of a formal requirements elicitation process with experts, SMAI was designed considering the clinical practice adopted in NAI and the geriatricians’ expertise, coordinators of the service. We believe that the development of the customized application, from the beginning, considering the context of the patient group helped achieve the stated objectives. In general, caregivers of the elderly have a very difficult journey, which includes different types of care. The major challenge involved in developing this application was not turning it into an extra activity, improving adherence to treatment protocols, as stressed by Arif et al. [16], and Fisher et al. [15].

The scope of the study developed by Wasilewski et al. in [46] is closely related to our proposal, focusing on family caregivers. The study points out that caregivers were satisfied with the usability and accessibility of the applications but usage was generally low and declined over time. In our evaluations we also verified that the usage declines over time, but not for all caregivers and that this depends on how the health team responds to the information sent by the caregiver.

The practice of performing small evaluations to refine the system pointed to problems in some features and interfaces that could be quickly modified in order provide its users more motivating activities, is aligned with the recommendations stressed in [47]. Technical problems, spanning from communication and database access to clock synchronization, were monitored in a daily-base so they could be promptly corrected. Informal feedbacks and more formal results captured in the assessments and evaluations were first internalized by the NAI health team in their meetings and then discussed with the computing team. The appropriate solutions were then applied. Some changes had to be reflected in both applications, SMAI Caregiver and SMAI Doctor as, for example, when the Glucose unit of measurement was changed. Thus the modifications were grouped in three types:

**Technical**, regarding improvements in the software architecture, communication and database access. For instance, from Stage 3 to Stage 4, when the number of users scaled from 10 caregivers to 30 caregivers (and 9 professionals), a full dashboard update was taking up to 3 min. This was optimized to 20 sec. Automatic image compression download was another implemented technical change.

**Functional,** which were implemented according to the health team requests. As mentioned, daily reports were reorganized into separate tabs: *general information*, *eating*, *bowel habits*, *urine*, *cough*, *eating problems* improving usability and report visualization. Some data input functions were modified to be context aware, as in the case of the *Temperature*, which began to pop a *Urination problems* panel if the patient had a fever. Another functional change example was directing the caregiver to send a text message, after sending a *NAI Alarm*, to help the health team with more information. Other minor changes were also implemented, such as more information options in the medication reminder. In addition to informing that given medicine should be given every 8 h, it was now possible to inform it should be taken with breakfast, lunch or dinner.

**Interface,** regarding improved forms of interaction with the applications. For instance a notification with a blinking NAI icon on screen, reminding something needed attention was introduced in SMAI Caregiver. A quick view of last caregiver interventions with a colored border for each patient on the dashboard was introduced in SMAI Doctor. As a last example, the list of exchanged text messages was improved with a conversation style display.

Tables 8 and 9 summarize important improvements and the project stages when they were implemented.

**Table 8:** SMAI Caregiver application main improvements

Stg. 2	Blinking NAI icon on screen, reminding something needs attention.
Stg. 3	Dosage of the medicine with more adjustment options; Unit measure for Glucose dosage Mmol/L → mg/dL;
	“How do you feel” report changed according to feedback, great/good/regular → normal/tired/stressed;
	Report separated in categories.
Stg. 4	Button with confirmation on caregiver report;
	Remedies intake reminder with coffee/lunch/dinner
	and “continuous use”.

**Table 9:** SMAI Doctor application main improvements

Stg. 2	Automatic download of images;
	Add/delete new patients on the Dashboard.
Stg. 3	Dashboard update more scalable and efficient;
	Medication reminder with fine grained dosage adjustment;
	Quick view of the last interactions of the caregiver (colored border of each patient on the dashboard);
	Notification when a caregiver views an appointment;
	Included a preloaded list of all medications;
	Change in the text message list with a chat style display.
Stg. 4	Easier account creation for health professionals.

From the Focus Group interviews, it was common sense among caregivers that the use of the application gave them the sensation of being connected to the health team. They also commented on receiving text messages sent by a professional of the health team in response to a previously sent message or alarm. Even if the text messages were not immediate, they always contained important care advice and were comforting. Some have mentioned having received messages with care advice, although they had not required or sent messages or alerts. This was due to the monitoring routines adopted by the NAI health team, with daily evaluations of the information and reports sent, allowing more effective support to the group of caregivers and avoiding discontinuation in using the system as pointed out by Nicholas et al. [24] and Lee et al. [25].

## Conclusion

SMAI has a modular architecture, facilitating the addition of new features or modifications. Non-functional features such as connectivity fault tolerance and security aspects have been implemented.

In addition, Android SMAI Caregiver and SMAI Doctor applications have low data consumption. In order to prevent intense access to the SMAI server, periodic activities such as sending location and checking new information may have a customized period of time and employ a specific prefetch mechanism. These non-functional aspects are important to make the system scalable.

In general, the use of SMAI represented a positive change for the family caregivers and for the NAI health team. The provision of remote health care, also called *health care delivery*, was perceived as a new paradigm in the interaction model between patient and health professional.

SMAI applications have been used for more than three years. The system is continuously updated with suggestions that emerge from the feedback of health professionals and caregivers. The software is registered with the Brazilian INPI (reg. BR 51 2015 000668 1).

## Data Availability

Raw data collected during the 18 months are being kept in the custody of the Rio de Janeiro State University and are available upon request. SMAI Caregiver *app* is not publicly available yet, but can be made available to alfa-testers given a NDA is signed.

## Abbreviations

A: Agree
App: Software application, usually for mobile phones
CI: Confidence interval
D: Disagree
eHealth: Electronic health
ISO: International organization for standardization
LB: Lower bound for a 95% confidence interval
LCC: Computer science laboratory
M: Weighted mean
N: Neutral
NAI: Care center for the elderly (*Núcleo de Atenção ao Idoso*)
SA: Strongly agree
SD: Strongly disagree
SDV: Standard deviation
SMAI: Mobile system for elderly monitoring (*Sistema Móvel de Assistência ao Idoso*)
SMS: Short message system
SUS: System usability scale
UB: Upper bound for a 95% confidence interval
UERJ: Rio de Janeiro State University (*Universidade do Estado do Rio de Janeiro*)
